# Supplementary material for: Replacing Animal-Based Proteins with Plant-Based Proteins Changes the Composition of a Whole Nordic Diet—A Randomised Clinical Trial in Healthy Finnish Adults
Source: Nutrients. 2020 Mar 28;12(4):943. doi: 10.3390/nu12040943 (PMC7231027; doi:10.3390/nu12040943)
Supplement: Supplementary file 1 [file nutrients-12-00943-s001.zip › Supplements_Pajari/Supplementary Table 1.docx]

**Supplementary Table 1.** Nutrient intake in the intervention groups at the baseline (for all nutrients *p*>0.05) and at the end of the 12-week intervention period.

|  | **ANIMAL** | | **50/50** | | **PLANT** | | ***p* (ANOVA)** |
| --- | --- | --- | --- | --- | --- | --- | --- |
|  | **Baseline (0 wk)** | **End (12 wk)** | **Baseline (0 wk)** | **End (12 wk)** | **Baseline (0 wk)** | **End (12 wk)** | **endpoint** |
| Energy intake (kJ) | 9124±2179 | 9222±1948 | 8731±1959 | 8653±1839 | 8788±1675 | 9096±1434 | 0.201 |
| Energy intake (kcal) | 2180±520 | 2204±465 | 2087±468 | 2068±439 | 2101±400 | 2174±343 | 0.200 |
| Protein (E%) | 18.5±3.2 | 18.2±3.1 | 17.3±3.1 | 16.9±2.2 | 17.7±3.4 | 15.2±2.0 | <0.001^1,2^ |
| Protein (g/day; food records) | 99.4±31.2 | 99.2±28.9 | 88.5±25.6 | 85.5±18.0 | 91.3±23.6 | 80.9±15.1 | <0.001^1,3^ |
| Protein (g/day; urinary N) | 93.1±29.0 | 99.3±26.2 | 85.9±20.1 | 86.5±22.6 | 93.8±24.1 | 77.5±12.4 | <0.001^1,3^ |
| Carbohydrates (E%) | 39.0±6.6 | 39.8±5.7 | 40.3±5.8 | 41.0±4.5 | 40.7±5.7 | 42.2±5.2 | 0.248 |
| Fat (E%) | 37.7±5.7 | 36.9±4.9 | 37.2±6.1 | 37.1±5.8 | 36.6±5.2 | 37.1±5.1 | 0.988 |
| Saturated fatty acids (E%) | 13.2±2.9 | 12.9±2.6 | 13.1±4.3 | 10.6±2.9 | 12.3±2.8 | 9.0±2.2 | <0.001^1,2,3^ |
| Monounsaturated fatty acids (E%) | 12.9±2.6 | 12.9±2.1 | 12.7±2.7 | 13.5±2.8 | 12.9±2.6 | 13.8±2.7 | 0.251 |
| Polyunsaturated fatty acids |  |  |  |  |  |  |  |
| Total (E%) | 6.2±2.0 | 6.1±1.3 | 6.3±1.5 | 8.0±1.7 | 6.6±1.8 | 9.5±1.8 | <0.001^1,2,3^ |
| n-6 PUFAs (E%) | 4.6±1.4 | 4.4±1.0 | 4.6±1.2 | 5.7±1.5 | 4.7±1.3 | 7.1±1.3 | <0.001^1,2,3^ |
| n-3 PUFAs (E%) | 1.7±0.7 | 1.6±0.4 | 1.6±0.6 | 1.9±0.5 | 1.7±0.6 | 2.2±0.5 | <0.001^1,3^ |
| α-linolenic acid (E%) | 1.1±0.5 | 1.2±0.3 | 1.1±0.4 | 1.5±0.5 | 1.1±0.4 | 1.5±0.4 | <0.001^1,3^ |
| Cholesterol (mg/d) | 315±15 | 278±73 | 294±129 | 192±72 | 282±127 | 139±61 | <0.001^1,2,3^ |
| Fibre (g) | 30.0±12.3 | 28.9±13.1 | 28.4±8.7 | 34.7±10.7 | 27.7±8.4 | 37.0±7.0 | <0.001^1,3^ |
| Fibre (g/MJ) | 3.3±1.1 | 3.1±0.8 | 3.3±0.9 | 4.0±0.9 | 3.2±0.8 | 4.1±0.6 | <0.001^1,3^ |

N nitrogen. *P* values for log10-transformed intakes. ^1^*p*>0.05 between ANIMAL and PLANT, ^2^*p*<0.05 between 50/50 and PLANT, ^3^*p*<0.05 between ANIMAL and 50/50, in Bonferroni comparison
