# Supplementary material for: Replacing Animal-Based Proteins with Plant-Based Proteins Changes the Composition of a Whole Nordic Diet—A Randomised Clinical Trial in Healthy Finnish Adults
Source: Nutrients. 2020 Mar 28;12(4):943. doi: 10.3390/nu12040943 (PMC7231027; doi:10.3390/nu12040943)
Supplement: Supplementary file 1 [file nutrients-12-00943-s001.zip › Supplements_Pajari/Supplementary Table 2.docx]

| **Food group** | ***Energy*** | | | ***Carbohydrates*** | | | ***Fibre*** | | |
| --- | --- | --- | --- | --- | --- | --- | --- | --- | --- |
|  | ANIMAL | 50/50 | PLANT | ANIMAL | 50/50 | PLANT | ANIMAL | 50/50 | PLANT |
| Meat dishes | **20.8 ± 7.9** | **11.7 ± 5.7** | 5.8 ± 4.4 | **12.0 ± 7.9** | 5.2 ± 3.9 | 4.6 ± 4.9 | 7.6 ± 5.1 | 4.1 ± 3.1 | 2.5 ± 2.8 |
| Milk and dairy products | **15.0 ± 5.2** | 8.1 ± 4.8 | 4.1 ± 3.0 | 10.3 ± 6.5 | 4.9 ± 3.2 | 2.5 ± 2.3 | 1.5 ± 4.4 | 0.5 ± 1.3 | 0.3 ± 0.8 |
| Fish dishes | 3.7 ± 2.3 | 3.9 ± 3.3 | 4.0 ± 2.9 | 2.3 ± 1.9 | 2.5 ± 2.7 | 2.2 ± 2.1 | 1.2 ± 1.0 | 1.1 ± 1.3 | 0.9 ± 0.8 |
| Egg dishes | 1.5 ± 1.7 | 1.4 ± 1.6 | 0.9 ± 1.2 | 0.0 ± 0.0 | 0.1 ± 0.2 | 0.2 ± 0.8 | 0.0 ± 0.0 | 0.1 ± 0.8 | 0.2 ± 1.0 |
| Cereals and bakery products | **29.6 ± 7.7** | **31.1 ± 8.3** | **31.8 ± 9.8** | **44.7 ± 10.5** | **45.6 ± 10.1** | **44.6 ± 11.2** | **54.4 ± 10.8** | **48.0 ± 11.0** | **44.4 ± 10.7** |
| Vegetables and vegetable dishes | 5.8 ± 3.5 | **15.0 ± 7.6** | **19.1 ± 7.8** | 5.8 ± 3.8 | **12.9 ± 6.9** | **15.9 ± 7.1** | **14.4 ± 7.9** | **26.3 ± 10.6** | **28.8 ± 10.3** |
| Nuts and seeds | 1.1 ± 1.9 | 2.6 ± 3.5 | **7.6 ± 4.5** | 0.2 ± 0.4 | 0.3 ± 0.4 | 1.5 ± 1.2 | 1.3 ± 2.2 | 1.8 ± 2.4 | 4.4 ± 3.3 |
| Plant-based dairy-like products | 0.0 ± 0.2 | 2.1 ± 2.7 | 4.2 ± 4.9 | 0.0 ± 0.2 | 2.2 ± 3.7 | 4.1 ± 5.3 | 0.0 ± 0.3 | 2.2 ± 4.0 | 3.4 ± 4.6 |
| Potatoes and potato dishes | 2.1 ± 2.5 | 1.8 ± 3.0 | 0.9 ± 1.6 | 3.0 ± 3.7 | 2.4 ± 3.9 | 1.0 ± 1.6 | 1.6 ± 2.0 | 1.4 ± 4.0 | 0.5 ± 0.9 |
| Fruits, berries, fruit and berry dishes | 6.8 ± 3.5 | 7.5 ± 4.7 | 7.1 ± 4.2 | **12.5 ± 6.5** | **13.2 ± 7.7** | **12.9 ± 8.3** | **16.4 ± 7.2** | **12.6 ± 7.7** | **12.0 ± 6.2** |
| Beverages | 3.4 ± 3.2 | 3.6 ± 8.0 | 4.2 ± 2.9 | 2.6 ± 2.6 | 3.9 ± 9.0 | 3.5 ± 3.1 | 0.0 ± 0.2 | 0.3 ± 1.4 | 0.1 ± 0.2 |
| Sugar and confectionery | 3.6 ± 4.1 | 3.8 ± 3.9 | 2.9 ± 4.3 | 5.7 ± 7.3 | 5.5 ± 5.6 | 4.9 ± 8.2 | 0.9 ± 1.3 | 0.9 ± 1.3 | 0.3 ± 0.6 |
| Miscellaneous ^1^ | 0.7 ± 1.5 | 0.9 ± 2.0 | 1.6 ± 2.2 | 0.6 ± 0.9 | 1.0 ± 1.9 | 1.7 ± 2.1 | 0.6 ± 1.0 | 0.4 ± 0.8 | 1.7 ± 2.7 |
| Fat spreads, oils and dressings | 5.9 ± 3.7 | 6.5 ± 4.6 | 5.8 ± 4.4 | 0.3 ± 0.4 | 0.3 ± 0.5 | 0.4 ± 1.2 | 0.1 ± 0.2 | 0.3 ± 0.8 | 0.5 ± 1.5 |

**Supplementary Table 2.** Sources of energy, carbohydrates and fibre in the intervention diets presented as average proportions (% ± SD) based on 4-day food record data. Three major sources of energy, carbohydrates and fibre are shown in bold.

^1^ Food group “Miscellaneous” includes dried fruits and berries, snacks, spices, piquant sauces, weight loss products, meal replacements, protein powders, protein bars and other miscellaneous foods.
