# Supplementary material for: Replacing Animal-Based Proteins with Plant-Based Proteins Changes the Composition of a Whole Nordic Diet—A Randomised Clinical Trial in Healthy Finnish Adults
Source: Nutrients. 2020 Mar 28;12(4):943. doi: 10.3390/nu12040943 (PMC7231027; doi:10.3390/nu12040943)
Supplement: Supplementary file 1 [file nutrients-12-00943-s001.zip › Supplements_Pajari/Supplementary Table 3.docx]

| **Food group** | ***Total Fat*** |  |  | ***SFA*** |  |  | ***PUFA*** |  |  | ***n-3 PUFA*** | | |
| --- | --- | --- | --- | --- | --- | --- | --- | --- | --- | --- | --- | --- |
|  | ANIMAL | 50/50 | PLANT | ANIMAL | 50/50 | PLANT | ANIMAL | 50/50 | PLANT | ANIMAL | 50/50 | PLANT |
| Meat dishes | **25.8 ± 9.7** | 15.0 ± 10.6 | 6.1 ± 5.5 | **24.4 ± 9.5** | **17.2 ± 11.7** | 8.7 ± 8.5 | **22.6 ± 10.8** | 10.9 ± 9.3 | 3.7 ± 3.7 | **19.1 ± 10.2** | 9.3 ± 9.7 | 2.8 ± 2.6 |
| Milk and dairy products | **17.1 ± 7.8** | 10.3 ± 7.6 | 5.7 ± 5.0 | **29.9 ± 12.9** | **21.0 ± 12.4** | 14.1 ± 11.8 | 3.2 ± 1.9 | 1.5 ± 1.6 | 0.8 ± 0.8 | 4.1 ± 4.4 | 1.9 ± 2.8 | 0.7 ± 0.9 |
| Fish dishes | 4.4 ± 2.9 | 4.5 ± 3.8 | 5.0 ± 4.1 | 3.2 ± 2.3 | 3.9 ± 3.5 | 5.7 ± 5.3 | 7.5 ± 5.3 | 6.4 ± 5.5 | 5.1 ± 4.4 | 16.3 ± 11.3 | **16.3 ± 13.4** | 12.9 ± 9.6 |
| Egg dishes | 2.9 ± 3.3 | 2.7 ± 3.1 | 1.5 ± 1.8 | 1.8 ± 2.1 | 2.0 ± 2.2 | 1.4 ± 1.8 | 2.7 ± 3.6 | 2.2 ± 3.4 | 0.8 ± 1.0 | 2.1 ± 3.4 | 1.8 ± 2.6 | 0.7 ± 1.1 |
| Cereals and bakery products | **18.0 ± 8.1** | **17.6 ± 7.3** | **20.0 ± 9.3** | 13.5 ± 10.3 | 14.2 ± 9.8 | **18.8 ± 11.1** | **28.2 ± 10.5** | **23.2 ± 8.2** | **22.9 ± 10.3** | **23.2 ± 11.0** | **19.6 ± 8.4** | **18.5 ± 8.7** |
| Vegetables and vegetable dishes | 5.8 ± 4.7 | **16.3 ± 9.6** | **19.8 ± 9.0** | 4.8 ± 4.9 | 10.9 ± 8.5 | **14.3 ± 8.1** | 7.5 ± 7.2 | **22.7 ± 12.1** | **23.6 ± 10.2** | 8.6 ± 7.3 | **22.5 ± 13.5** | **24.9 ± 11.9** |
| Nuts and seeds | 2.0 ± 3.5 | 5.2 ± 6.5 | **15.3 ± 8.4** | 0.7 ± 1.4 | 1.8 ± 2.9 | 7.6 ± 4.1 | 3.6 ± 5.9 | 10.2 ± 12.4 | **21.4 ± 12.6** | 3.0 ± 5.6 | 5.1 ± 8.6 | **17.5 ± 12.8** |
| Plant-based dairy-like products | 0.1 ± 0.3 | 2.3 ± 2.2 | 4.8 ± 5.2 | 0.0 ± 0.1 | 1.0 ± 1.3 | 3.0 ± 5.0 | 0.1 ± 0.9 | 2.8 ± 3.2 | 4.7 ± 6.9 | 0.1 ± 0.9 | 2.8 ± 3.6 | 3.9 ± 5.3 |
| Potatoes and potato dishes | 1.9 ± 3.1 | 1.9 ± 3.5 | 1.0 ± 2.4 | 1.8 ± 4.3 | 1.7 ± 3.3 | 1.0 ± 2.3 | 2.4 ± 4.2 | 2.3 ± 5.6 | 1.0 ± 2.9 | 1.9 ± 2.7 | 2.0 ± 4.4 | 0.8 ± 2.2 |
| Fruits, berries, fruit and berry dishes | 2.2 ± 2.6 | 2.6 ± 3.5 | 2.0 ± 3.2 | 1.2 ± 2.1 | 2.0 ± 3.0 | 1.4 ± 2.6 | 2.0 ± 1.7 | 1.7 ± 1.8 | 1.1 ± 1.3 | 2.4 ± 1.8 | 2.1 ± 2.1 | 1.5 ± 1.3 |
| Beverages | 0.7 ± 0.8 | 0.6 ± 0.6 | 0.7 ± 0.8 | 0.5 ± 1.4 | 0.3 ± 1.1 | 0.6 ± 2.1 | 0.1 ± 0.1 | 0.1 ± 0.3 | 0.0 ± 0.1 | 0.1 ± 0.3 | 0.3 ± 1.0 | 0.1 ± 0.2 |
| Sugar and confectionery | 2.6 ± 2.9 | 3.6 ± 4.2 | 1.6 ± 2.7 | 4.1 ± 4.6 | 6.6 ± 7.4 | 3.8 ± 6.2 | 0.5 ± 0.6 | 0.6 ± 0.9 | 0.2 ± 0.4 | 0.2 ± 0.2 | 0.3 ± 0.9 | 0.1 ± 0.2 |
| Miscellaneous ^1^ | 1.0 ± 3.2 | 1.1 ± 3.0 | 2.0 ± 3.8 | 0.5 ± 1.7 | 0.9 ± 3.0 | 1.6 ± 3.2 | 1.6 ± 5.5 | 0.9 ± 2.5 | 2.6 ± 6.1 | 1.6 ± 5.3 | 0.9 ± 2.6 | 2.2 ± 5.3 |
| Fat spreads, oils and dressings | 15.5 ± 9.3 | **16.3 ± 10.6** | 14.5 ± 10.7 | **13.6 ± 9.7** | **16.5 ± 11.2** | **18.0 ± 14.5** | **18.0 ± 12.9** | **14.5 ± 10.5** | 12.1 ± 10.0 | **17.3 ± 13.4** | 15.1 ± 13.0 | 13.4 ± 11.4 |

**Supplementary Table 3.** Sources of total fat, saturated fatty acids (SFA), polyunsaturated fatty acids (PUFA) and n-3 polyunsaturated fatty acids (n-3 PUFA) in the intervention diets presented as average proportions (% ± SD) based on 4-day food record data. Three major sources of total fat and each type of fatty acids are shown in bold.

^1^ Food group “Miscellaneous” includes dried fruits and berries, snacks, spices, piquant sauces, weight loss products, meal replacements, protein powders, protein bars and other miscellaneous foods.
